# Supplementary material for: Local and global crosstalk among heterochromatin marks drives DNA methylome patterning in Arabidopsis
Source: Nat Commun. 2022 Feb 14;13:861. doi: 10.1038/s41467-022-28468-5 (PMC8844080; doi:10.1038/s41467-022-28468-5)
Supplement: Supplementary file 1 — Supplementary Information [file 41467_2022_28468_MOESM1_ESM.pdf]

Local and global crosstalk among heterochromatin marks drives DNA methylome patterning in Arabidopsis

To *et al.*

Supplementary Fig. 1

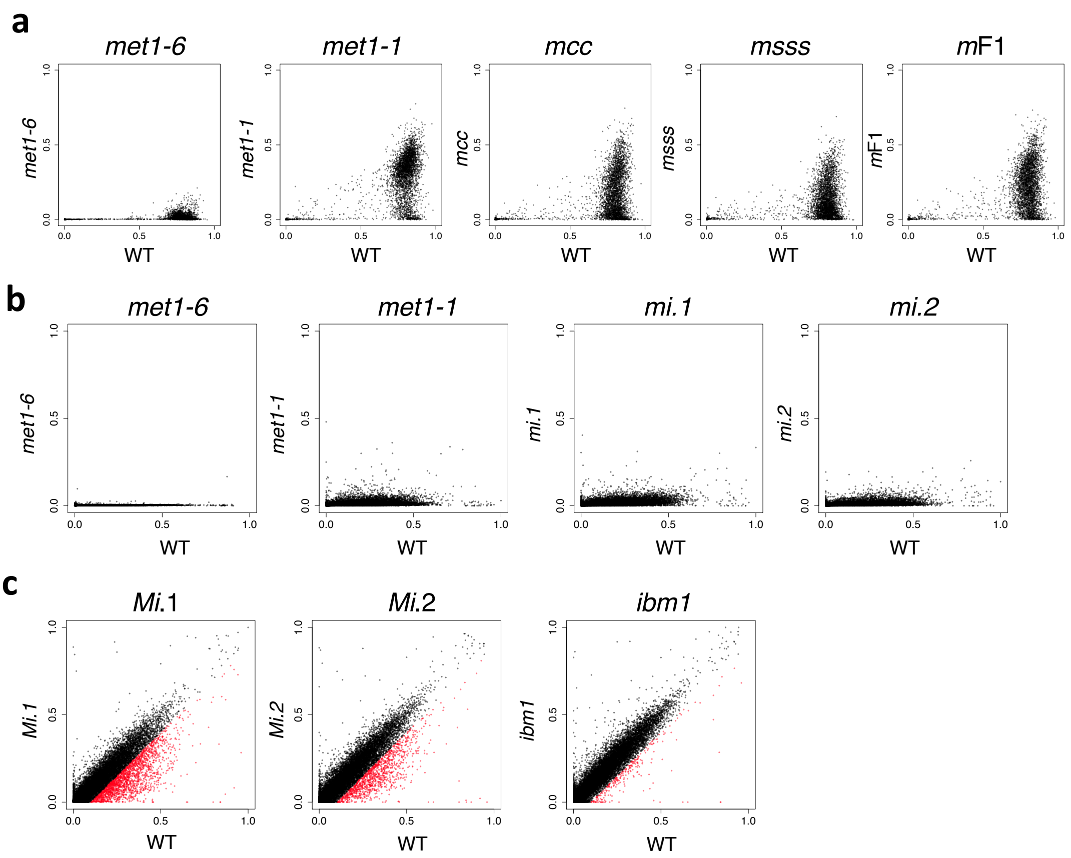

Supplementary Fig. 1 Loss of mCG in the *met1* mutants.

a The mCG levels for each TE genes in *met1-1* mutant compared to those of a wild-type (WT) plant. Results of *met1*-6, a null mutant of *MET1*, are shown in the left for comparison (GSE148753). **b** The mCG level compared between the *met1* mutants and WT for each of protein coding genes. **c** Loss of mCG detected in two individual *Mi* (*MET1/MET1 ibm1/ibm1* progeny originated from *MET1/met1-1 IBM1/ibm1* double heterozygote; as shown in Fig. 3a). mCG level of each protein coding genes in *Mi* plants compared to that in WT. Genes with mCG loss (WT - *Mi* >0.1) are shown in red. The *ibm1* mutant plant without experiencing *MET1* heterozygous state did not show such extensive loss of mCG (right panel).

Supplementary Fig. 2

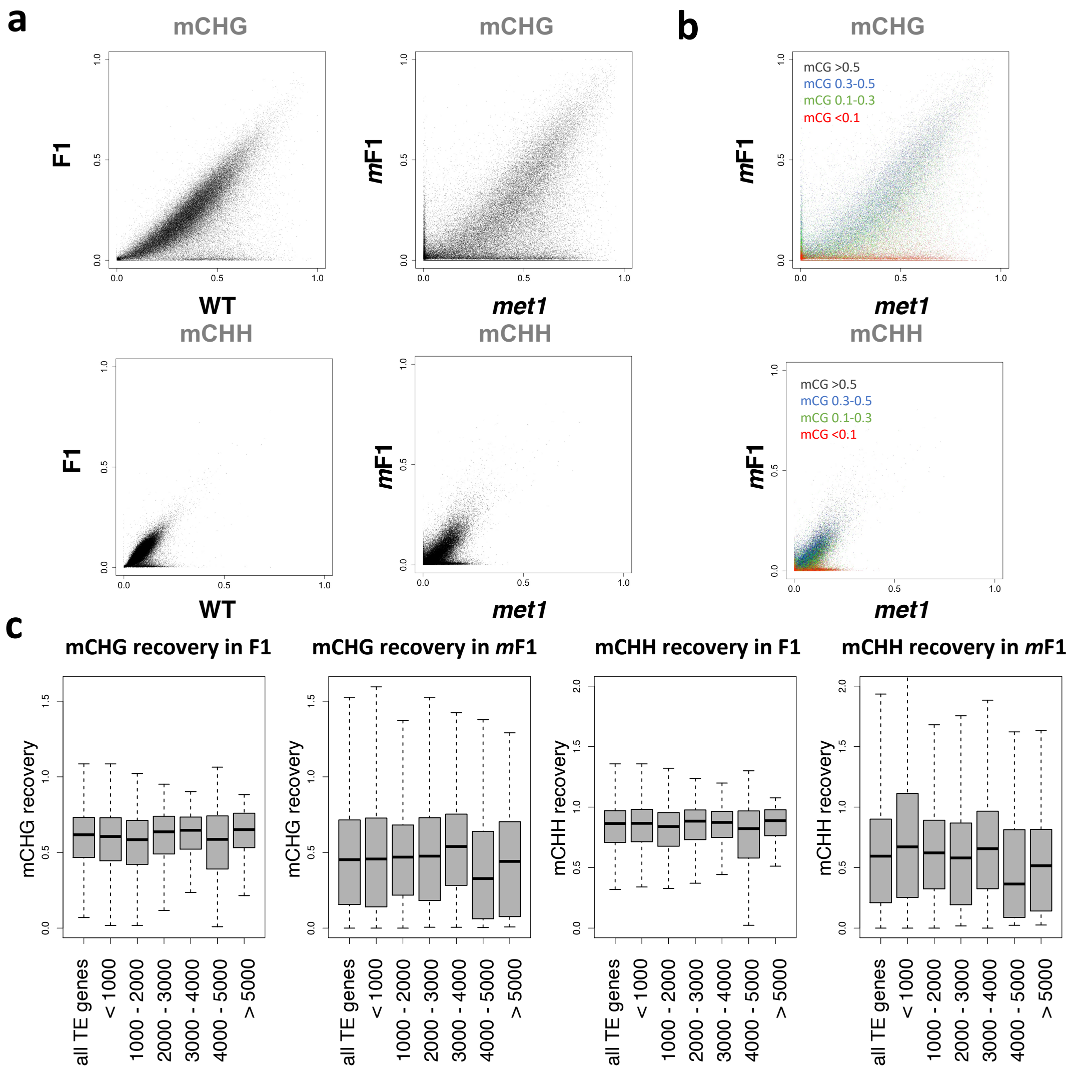

Supplementary Fig. 2 mCH recovers where mCG is present.

a mCH levels in the F1 (left) and *m*F1 (right) were analyzed in 100-bp bins and compared with that of WT and *met1*, respectively. **b** Comparison of mCH levels in the *m*F1 and *met1* in 100-bp bins colored according to the mCG levels in *m*F1 (Red : <0.1, green : 0.1–0.3, blue : 0.3–0.5, and black : >0.5 for mCG in *m*F1). **c** The efficiency of mCH recovery in TE genes were compared with their length. The efficiency of recovery was calculated as F1 / WT or *m*F1 / *met1*, respectively. To avoid division by values near zero, TE genes with mCHG (>0.1) or with mCHH (>0.03) in both WT and *met1* mutant were used (for mCHG: n=3212, for mCHH: n=2915). Outliers are not shown. The center line and box edges represent quartiles and whiskers range 1.5 times of the interquartile from the box edges.

Supplementary Fig. 3

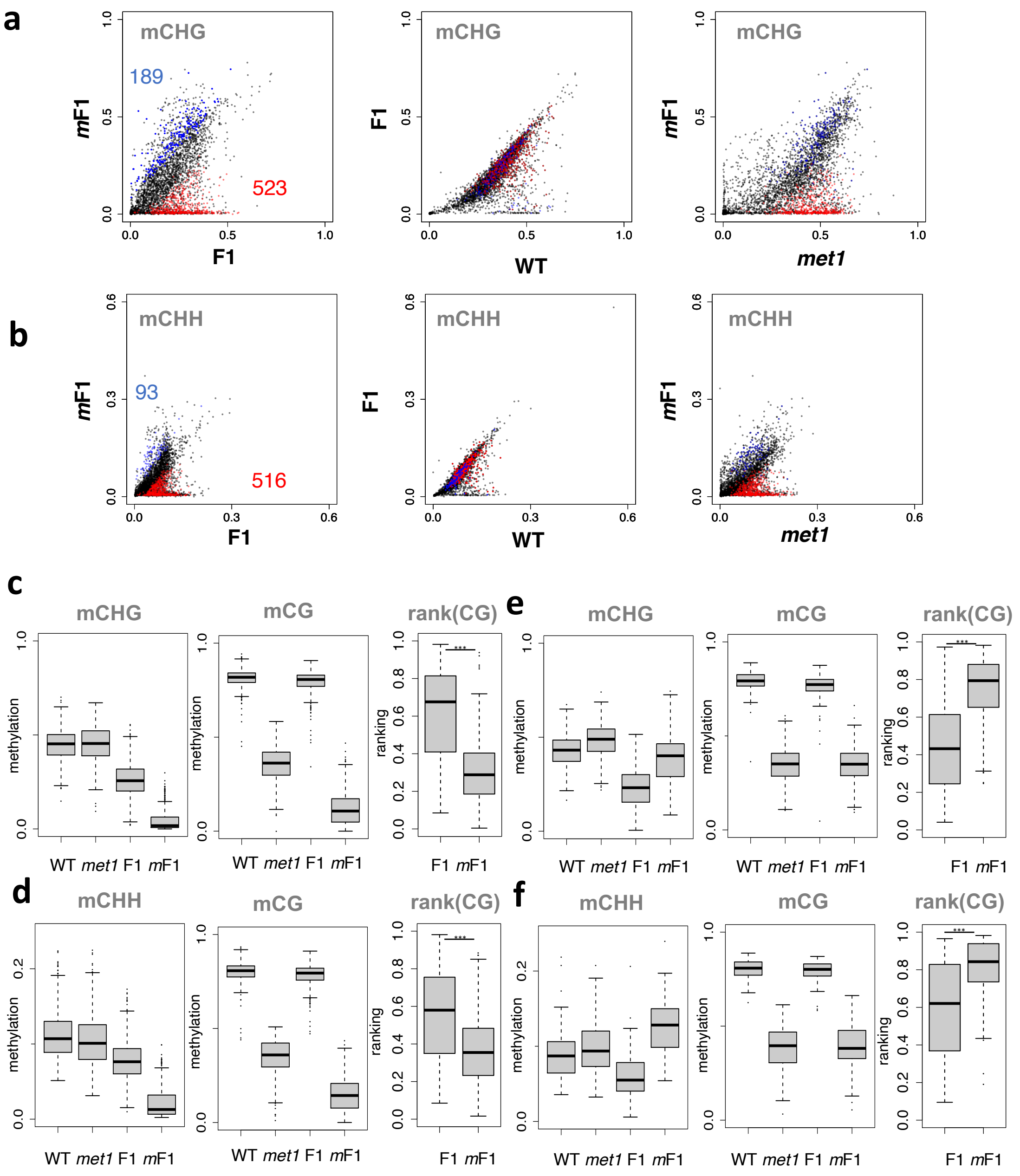

Supplementary Fig. 3 mCH recovers more efficiently in TE genes with relatively higher mCG levels within the genome.

a TE genes statistically and significantly higher (blue, n=189) and lower (red, n=523) mCHG levels in *m*F1 than in F1 (see Methods for detail). **b** TE genes statistically and significantly higher (blue, n=93) and lower (red, n=516) mCHH levels in *m*F1 than in F1. **c,d** TE genes with inefficient mCH recovery in *m*F1 (red in **a**

(n=523) or **b** (n=516), respectively) compared for their mCH (left), mCG (middle), as well as their mCG level ranking among all TE genes (rank(CG) panel in right). The ranking was normalized into the range from 0 (bottom) to 1 (top). **e,f** TE genes with more efficient mCH recovery in *m*F1 than in F1 (blue in **a** (n=189) or **b** (n=93), respectively) compared for their mCH (left), mCG (middle), as well as their mCG level ranking among all TE genes (rank(CG) panel in right). The ranking was normalized into the range from 0 (bottom) to 1 (top). Note that, despite of much lower mCG levels in *m*F1 than in F1, the ranking of mCG among all TE genes are significantly higher in *m*F1, which is concomitant with higher mCH levels in *m*F1 than in F1. (***$P < 0.0001$, Wilcoxon signed rank test, two-sided). In addition, the lower mCG levels in *m*F1 than in *met1-1* suggests the role of mCH for the maintenance of mCG for these TE genes in the *met1-1* mutant background. In boxplots (**c–f**), the center line and box edges represent quartiles and whiskers range 1.5 times of the interquartile from the box edges.

Supplementary Fig. 4

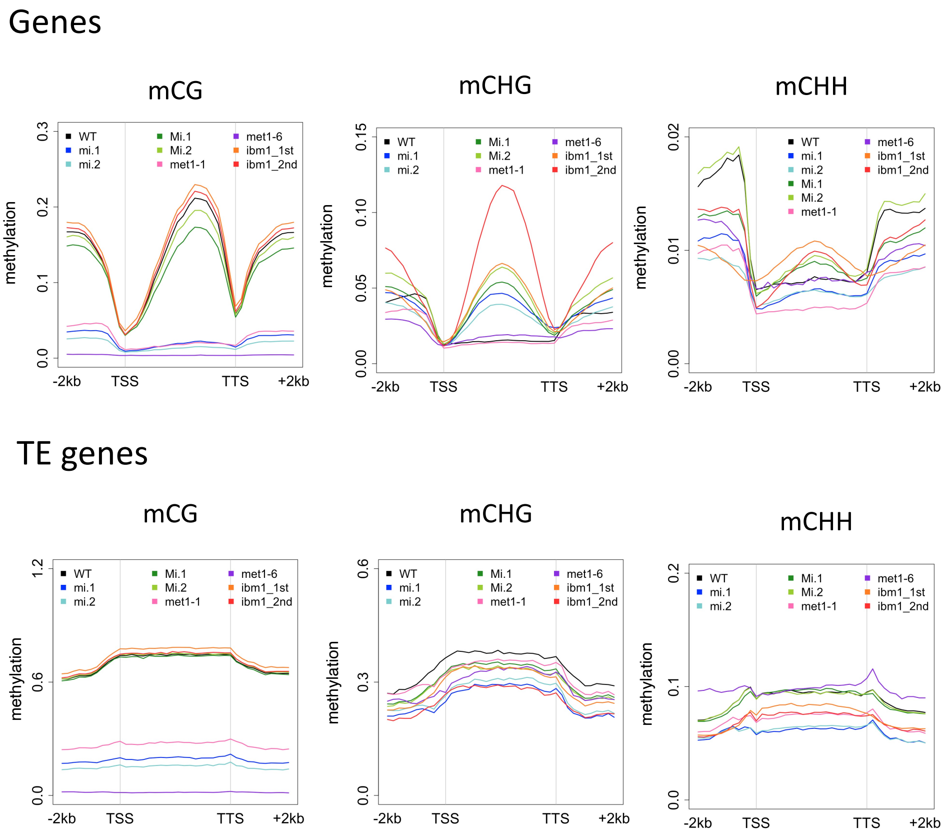

Supplementary Fig. 4 mCG and mCH patterns over genes and TE genes.

Averaged mCG (left), mCHG (middle) and mCHH (middle) over genes and TE genes. In each genotype, mean values are shown. To exclude the mis-annotated TEs, the genes with mCHG in the WT (>0.05) are excluded from the analysis for genes (excluded n=1562; analyzed n=26723).

Supplementary Fig. 5

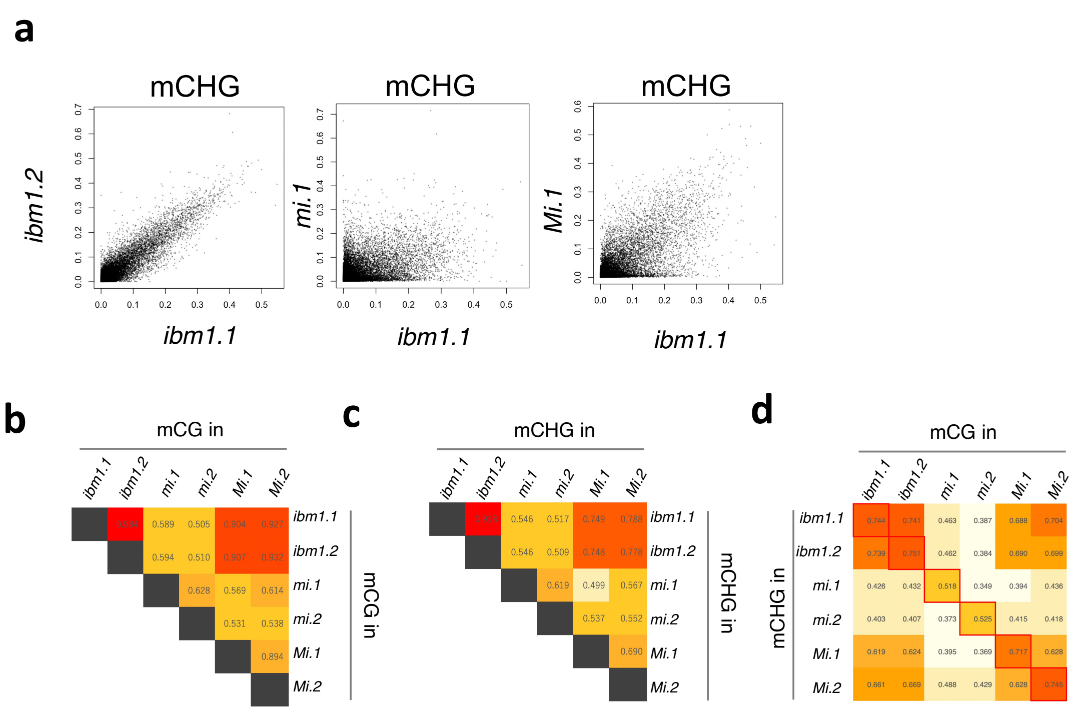

Supplementary Fig. 5 The spectrum of genic mCH is different between *ibm1* and *mi*.

a Comparison of mCHG levels in the indicated genotypes. Spectrum of mCHG differs between *ibm1* and *mi*. **b** Correlation of mCG between the indicated individuals. The number in the box represents the pearson's correlation coefficient. **c** Correlation of mCHG between the indicated individuals. The number in the box represents the pearson's correlation coefficient. **d** Correlation between mCHG and mCG in the indicated individuals. The best correlated in the individuals are boxed in red. The number in the box represents the pearson's correlation coefficient. To exclude the mis-annotated TEs, the genes with mCHG in the WT (>0.05) are excluded from the analysis in panels (**a**)–(**d**) (excluded n=1562; analyzed n=26723).

Supplementary Fig. 6

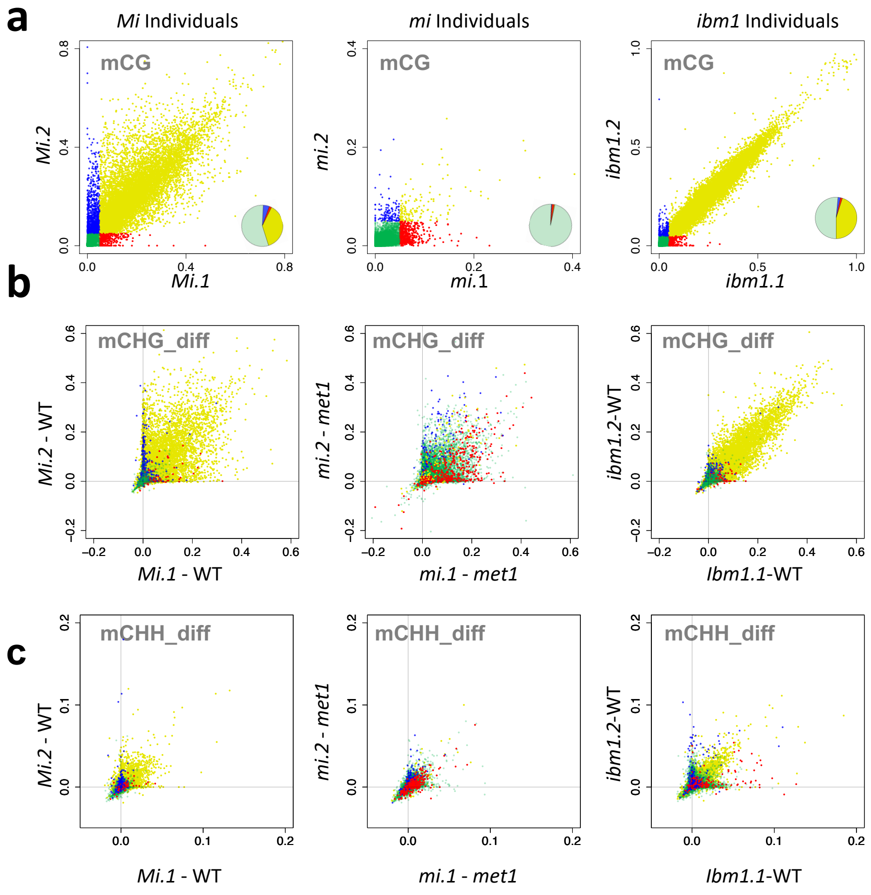

Supplementary Fig. 6 Genic mCH is correlated with the presence of mCG in the individual *mi* and *ibm1* plants.

a Differential mCG levels of genes in the two *mi* and *ibm1* individuals. The genes with the mCG presence (>0.05) or absence (<0.05) in the individuals are colored yellow (commonly CG methylated), green (commonly CG hypomethylated), red (CG methylated only in *mi*.1 individual) and blue (CG methylated only in *mi*.2 individual). The ratio of each group was shown as pie chart. **b** The ectopic mCHG in genes in the *ibm1* individuals. The genes are colored according to the groups in (**a**). To exclude the mis-annotated TEs, the genes with mCHG in the WT (>0.05) are excluded from the analysis (excluded n=1562; analyzed n=26723). **c** The ectopic mCHH in genes in the *ibm1* individuals. The genes are colored according to the groups in (**a**). To exclude the mis-annotated TEs, the genes with mCHH in the WT (>0.02) are excluded from the analysis (excluded n=1492; analyzed n=26849).

Supplementary Fig. 7

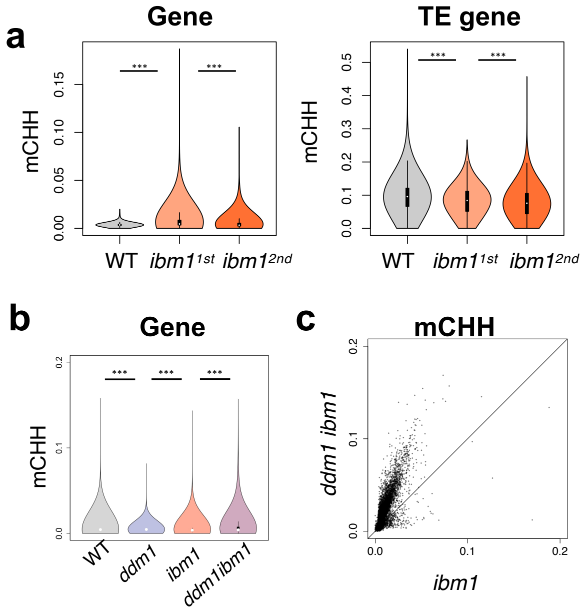

Supplementary Fig. 7 mCHH is controlled not only locally but also globally.

a Violin plots of mCHH in genes and TE genes (****P* < 0.0001, Wilcoxon signed rank test, two-sided). **b** Violin plot showing that *ddm1* mutation further enhanced the *ibm1*-induced accumulation of mCHH in genes (****P* < 0.0001, Wilcoxon signed rank test, two-sided). **c** The *ibm1*-induced genic mCHH was globally enhanced in *ddm1 ibm1* double mutant (line: y=x). To exclude the mis-annotated TEs, the genes with mCHH in the WT (>0.02) are excluded from the analysis in (**a**–**c**) (excluded n=1492; analyzed n=26849).
